# Connectivity Measures in EEG Microstructural Sleep Elements

**DOI:** 10.3389/fninf.2016.00005

**Published:** 2016-02-17

**Authors:** Dimitris Sakellariou, Andreas M. Koupparis, Vasileios Kokkinos, Michalis Koutroumanidis, George K. Kostopoulos

**Affiliations:** ^1^Neurophysiology Unit, Department of Physiology, University of PatrasPatras, Greece; ^2^Department of Clinical Neurophysiology and Epilepsy, Guy's and St. Thomas' NHS Foundation TrustLondon, UK; ^3^Division of Neuroscience, Department of Basic and Clinical Neuroscience, King's College LondonLondon, UK

**Keywords:** EEG-element connectivity, EEG microstructure, time-frequency analysis, imaginary part of coherence, sleep spindle connectivity

## Abstract

During Non-Rapid Eye Movement sleep (NREM) the brain is relatively disconnected from the environment, while connectedness between brain areas is also decreased. Evidence indicates, that these dynamic connectivity changes are delivered by microstructural elements of sleep: short periods of environmental stimuli evaluation followed by sleep promoting procedures. The connectivity patterns of the latter, among other aspects of sleep microstructure, are still to be fully elucidated. We suggest here a methodology for the assessment and investigation of the connectivity patterns of EEG microstructural elements, such as sleep spindles. The methodology combines techniques in the preprocessing, estimation, error assessing and visualization of results levels in order to allow the detailed examination of the connectivity aspects (levels and directionality of information flow) over frequency and time with notable resolution, while dealing with the volume conduction and EEG reference assessment. The high temporal and frequency resolution of the methodology will allow the association between the microelements and the dynamically forming networks that characterize them, and consequently possibly reveal aspects of the EEG microstructure. The proposed methodology is initially tested on artificially generated signals for proof of concept and subsequently applied to real EEG recordings via a custom built MATLAB-based tool developed for such studies. Preliminary results from 843 fast sleep spindles recorded in whole night sleep of 5 healthy volunteers indicate a prevailing pattern of interactions between centroparietal and frontal regions. We demonstrate hereby, an opening to our knowledge attempt to estimate the scalp EEG connectivity that characterizes fast sleep spindles via an “EEG-element connectivity” methodology we propose. The application of the latter, via a computational tool we developed suggests it is able to investigate the connectivity patterns related to the occurrence of EEG microstructural elements. Network characterization of specified physiological or pathological EEG microstructural elements can potentially be of great importance in the understanding, identification, and prediction of health and disease.

## Introduction

Sleep is a physiological process categorized in cycles, stages, and furthermore characterized by its microstructural aspects and elements, such as the K-complex and the sleep spindle. The microstructural architecture of EEG has previously been proven to play a crucial role in the understanding of the neurophysiological and functional aspects of sleep (Nicholas et al., 2002).

Sleep spindles are prominent EEG rhythms observed during non-rapid eye movement (NREM) sleep (Kim et al., 2012). They are oscillatory EEG activities in the sigma frequency band (~11–16 Hz) of fusiform morphology that last around 0.5–3 s (De Gennaro and Ferrara, 2003; Iber et al., 2007; Lüthi, 2013). Two types of sleep spindles have been recognized; fast spindles at 14–15 Hz maximal in centroparietal regions and slow spindles at 12–13 Hz predominant in frontal areas (Gibbs and Gibbs, 1950; Zeitlhofer et al., 1997). The spectral frequency of both types characterizes individual subjects and may be inherited (Van Dongen et al., 2005). Sleep spindles have been associated with cognitive faculties (Fogel and Smith, 2011) and intelligence (Knoblauch et al., 2003), with normal aging processes (Crowley et al., 2002) and various disease states (e.g., schizophrenia, Parkinson, Alzheimer, mental retardation, abnormal maturation) but also with recovery processes as in post brain stroke (De Gennaro and Ferrara, 2003; Ferrarelli et al., 2010; Urakami et al., 2012). Furthermore, It has been shown in animal studies that sleep spindles play a significant role in sensory-motor brain development (Khazipov et al., 2004) and in the induction of long-term potentiation (Rosanova and Ulrich, 2005). Moreover, spindles have long been recognized as gating sensory information in the thalamus and thus promoting sleep maintenance in a noisy environment (De Gennaro and Ferrara, 2003; Lüthi, 2013). Based on their close temporal coupling to hippocampal sharp-wave ripples and slow-oscillation up-states, sleep spindles are now considered to be significantly involved in sleep-dependent memory consolidation (Schabus et al., 2004; Diekelmann and Born, 2010). Furthermore, during NREM sleep, the brain is considered to be relatively disconnected from the environment mainly because thalamocortical neurons fall into a hyperpolarisation and rhythmic bursting mode (Steriade, 2003). Such activity leads to the generation of sleep spindles in the cortex. The inhibitory “gating” by spindles of exteroceptive (Dang-Vu et al., 2011) and interoceptive (Landis et al., 2004) sensory perception, preserves the sleep state and thus enables sleep's restorative role as well as memory consolidation unperturbed by any on-going activity outside the brain. Experiments in rats have shown that the response of prefrontal pyramidal cortical neurons following to hippocampal input stops during sleep spindles (Peyrache et al., 2011). This suggests that thalamic inputs in burst mode can recruit massive intracortical inhibition, which may itself produce a local differentiation of the cortical area concerned, regardless of the input source. Furthermore, it has been claimed that during NREM sleep there is a decrease in brain inter-area connectivity (Massimini et al., 2005). In view of the above, it could be interesting to further investigate the role played by sleep spindles in this interregional connectivity change to provide insights into the mechanisms underlying sleep maintenance and memory consolidation.

The already “octogenarian” study of spindles has been greatly accelerated in the digital era and most recently by the support of brain imaging and advanced quantitative EEG analysis tools for automatic detection, topographical, and spectral analysis at scalp and intracranial electrode space as well as non-invasive source localization (Dehghani et al., 2011; Frauscher et al., 2015). The latter reports show that in contrast to long-standing views of spindles as synchronous global events (Loomis et al., 1935; Contreras et al., 1997), scalp EEG spindles are generated by the asynchronous activity of diverse focal cortical sources rather than widespread synchronous oscillations. Furthermore, time-frequency correlation techniques reveal very interesting dynamics of spindle occurrence. Spindles occur throughout the episodes of NREM sleep, mostly apparent in NREM2 with temporal dynamics intra-cycle, i.e., appearing at NREM-REM transitions (Vyazovskiy et al., 2004; Halász and Bodisz, 2013) as well as across sleep cycles, i.e., appearing at later rather than earlier sleep cycles. They have a reciprocal relationship with delta activity (Dijk et al., 1993) and sustain effects of sleep deprivation, circadian factors and aging (De Gennaro and Ferrara, 2003). In addition, spindles are involved in trans-frequency grouping (Steriade, 2006) and modulation with both slow (Spoormaker et al., 2011), and gamma band cortical oscillations (Ayoub et al., 2012), and show a still cryptic association to hippocampal ripples (Clemens et al., 2011; Peyrache et al., 2011). They also have a very dynamic interaction with K-complexes (Kokkinos and Kostopoulos, 2011; Kokkinos et al., 2013).

Notwithstanding these advances, the cortical interaction networks that concern sleep spindles and their propagation amongst the various cortical areas still remain uncertain.

EEG connectivity measures that aim to assess the prevailing interconnected regional patterns of EEG records and include coherence (Nunez et al., 1997), directed transfer function (DTF; Kamiñski and Blinowska, 1991), partial directed coherence (Sameshima and Baccalá, 1999), synchronization likelihood (Stam and Van Dijk, 2002), phase lag index (Stam et al., 2007), imaginary part of coherence (Nolte et al., 2004) have been developed in the last years (David et al., 2004; De Vico Fallani et al., 2008).

Connectivity methodologies that focus on EEG microstructural elements of short duration (~s) have not yet been widely addressed and could possibly be able to provide with valuable insight with regards to underlying functional interregional mechanisms.

In this study, we describe a scalp EEG connectivity methodology designed for the investigation of microstructural EEG elements, as used in the development of a MATLAB-based tool. In this process, the topics of volume conduction, EEG reference problem and the directionality of information flow between cortical areas are being addressed.

We tested coherency and related estimates (i.e., Imaginary part of coherence) with respect to frequency, phase, directionality, the volume conduction, and EEG reference problems using artificially generated signals in order to provide with an overall proof-of-concept for the proposed approach. We furthermore test the role of a number of trials in the calculation of coherence with regards to statistical significance and suggest an error assessment approach for the suggested methodology.

Finally, we attempt to estimate the connectivity patterns formed due to the fast sleep spindle occurrence in five healthy subjects using a custom-built MATLAB-based connectivity tool.

## Methods

### Coherency and coherence estimates

Coherency is a widely used measure for characterizing linear dependence between a pair of stochastic processes as well as a quantitative measure of their phase consistency. Let *s*_*i*_(*f*) and *s*_*j*_(*f*) represent the complex Fourier transforms of two stochastic processes *x*_*i*_(*t*) and *x*_*j*_(*t*). For *N* epochs of the segmented time series, the cross-spectral density function *S*_*ij*_(*f*)of *i* and *j* for a single *n* epoch is defined as:

(1) $$\begin{array}{l} S_{ijn}\left(f\right)=s_{in}\left(f\right)s_{jn}^{*}\left(f\right) \end{array}$$

and the auto spectral density function (single epoch power spectrum) as:

(2) $$\begin{array}{l} S_{in}\left(f\right)=s_{in}\left(f\right)s_{in}^{*}\left(f\right) \end{array}$$

where ^*^ indicates the complex conjugate.

The cross-spectral density function estimation over *N* epochs is:

(3) $${\hat{S}}_{i\;j}\left(f\right)=\frac{1}{N}\sum_{n=\text{1}}^{N}S_{i\;jn}(f)$$

The coherency estimated upon *n* epochs is defined as the cross-spectrum normalized by the auto spectral density functions of *x*_*i*_(*t*) and *x*_*j*_(*t*):

(4) $$\begin{array}{l} {Ĉ}_{ij}\left(f\right)=\frac{\left|{Ŝ}_{ij}\left(f\right)\right|}{{\left({Ŝ}_{i}\left(f\right){Ŝ}_{j}\left(f\right)\right)}^{\frac{1}{2}}} \end{array}$$

Coherence is defined as the absolute value of coherency i.e., the normalized amplitude of the complex cross-spectrum number:

(5) $$\begin{array}{l} {Coh}_{ij}\left(f\right)=|{Ĉ}_{ij}\left(f\right)| \end{array}$$

**Figure A1:**
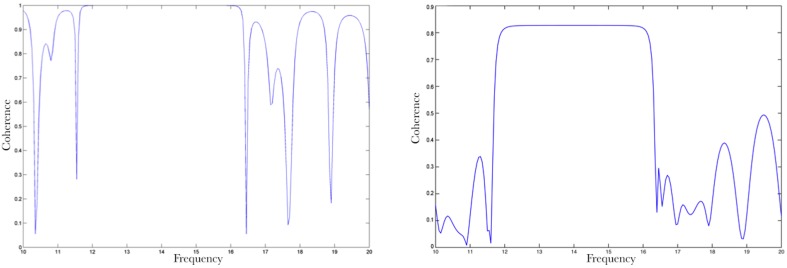
**Coherence between x and y signals: coherence values for 1 trial (left) Coherence values for 10 trials (right)**. The coherence values attributed to noise appear to be significantly reducing as the number of trials increase.

The expectation value for coherences can only be adequately estimated as an average over a sufficiently large number of epochs (**Figure A1**; Nunez et al., 1997). Conditionally, the confidence intervals depend on the normalized RMS error, which may be approximately given by (Equation 6).

(6) $$\begin{array}{l} e_{c^{2}}=\sqrt{2{∕}_{N}}\frac{1-{Ĉ}^{2}}{\left|Ĉ\right|} \end{array}$$

### Connectivity of microelements and the EEG microstructure

The main interest of connectivity studies is to estimate networks that generally characterize a subject or a group of subjects by averaging over long periods of recordings of multiple datasets of each subject or group of subjects.

Lack of extended bibliography and standardization for the estimation of connectivity for short duration EEG elements prompted us to initially test coherence and its imaginary part over artificially generated signals in MATLAB with respect to its basic well-known functions. Next, we investigate possible solutions for the volume conduction and EEG reference problems in order to propose a methodology for similar EEG studies. We hereby, show preliminary results for the sleep spindle related connectivity patterns as calculated in five subjects with the use of a custom MATLAB-based tool we developed in order to apply the proposed methodology.

We note here that in the case of “EEG-element connectivity,” we are interested in the dependence of connectivity as a function related to an identified EEG microstructural element in time and frequency.

### Methodology

The connectivity investigation of the EEG microstructure should ideally be able to determine levels of interregional interactions, determine the directionality of information flow and recognize significant results while dealing with the volume conduction and EEG reference problems at an adequate level.

At the tool-development level, investigation of the results over frequency and time should be accessible by global parameterization of the connectivity maps over frequency, time, and threshold domains. Moreover, easy adjustability for error assessment and statistical significance (α-level of significance) should also be provided.

#### Prepossessing for the element coherence estimation

The periodogram-inspired estimators of cross and power spectra for a pair of signals suffer from very high variance, which consequently causes the magnitude squared coherence (MSC) to fail. In order to calculate meaningful coherence estimates, averaging approaches such as Welch's method (Welch, 1967) are being used where the EEG recordings are being segmented into length L epochs, with *R* offset so that (*n* − 1)*R* + *L* ≤ *Q*, where *Q* is the total data length and *n* the number of segmented epochs. In this manner, a frequency resolution of *Df (Hz)* (Equation 7) is obtained for the MSC. Consequently, the increase in the number of epochs improves the coherence estimation accuracy as the resolution in the frequency domain drops.

(7) $$\begin{array}{l} Df=n∕L \end{array}$$

If only the signals are considered stochastic processes, then to further improve the coherence estimates one would average over multiple *K* periods in time repeating the above process in order to calculate an improved mean coherence value. Although the EEG signals can never be considered stationary, we proceed with the latter step as we focus on specific elements rather than whole EEG recordings: for the estimation of mean coherence *K* relatively identical element trials with well-defined and specific wave characteristics (i.e., amplitude, duration, frequency) are being selected. Hence, by being able to use the mean MSC we can more accurately estimate correlations with greater resolution over frequency and time. An Increased number of trials in the calculation drive the level of interaction and phase estimators of coherence to specifically relate with the element under investigation.

#### Volume conduction

Volume conduction in EEG recordings significantly affects coherence estimators. Electrical activity of the cortex disparately spreads across scalp electrodes at some distance from its generators allowing the same cortical activity to be measured by multiple neighboring electrodes at the same time i.e., with zero phases.

To exclude volume conducted EEG activity coherence values that refer to signal correlations with 0-lag phase are adjusted to zero. The fundamental assumption about 0-lag activity is that an observed scalp potential has no time lag to the underlying source activity, which is widely accepted (Stinstra and Peters, 1998). Therefore, we hypothesized here that volume conduction does not cause time lag, with the latter being only present in causal physiological processes. In theory, a neuron can send action potentials through recurrent collaterals to several equidistant neurons with conventionally zero time lags. In practice though, this is rather unlikely to happen in a large enough number of neurons that are recorded by the EEG sensors and differentiate among other asynchronous potentials.

This methodology employs the imaginary part of coherence (Nolte et al., 2004) as the estimator, which stands for the perpendicular presented to the real axis values of a complex number, positively increasing in magnitude to the left. In this way, when the correlated activity of a pair of signals is simultaneous i.e., has zero lag identical activity, the estimator's values are adjusted to zero. Such values can generally be attributed to volume conduction.

(8) $$\begin{array}{l} z=x+yi=re^{i\varphi }=r\left(cos\varphi +isin\varphi \right) \end{array}$$

(9) $$\begin{array}{l} r=|z|=\sqrt{x^{2}+y^{2}} \end{array}$$

(10) $$\begin{array}{l} \varphi =\text{arg}\left(z\right) \end{array}$$

#### Directionality of information flow

Coherency can also be considered as an estimator of synchronisations as Δφ = φ_ι_ − φ_*j*_ between the signals in channels *i* and *j* at a specific frequency that is essentially measured. The sign of the phase between the two compared signals can suggest possible directionality of the information flow between the two areas. For a pair of correlated signals at a specific frequency, the earlier activated one is considered as causal to the other, and vice versa. In general, if the imaginary part of the coherence is positive, then *x* and *y* are interacting and *x* is earlier than *y*, indicating that information is flowing from *x* to *y* (Nolte et al., 2004).

#### The EEG reference problem

Phase reversal in a sequential bipolar montage refers to the opposite and simultaneous deflection of pens in channels that contain a common electrode (Hirsch and Brenner, 2010).

The reference electrode is often positioned within the active array of electrodes especially in clinical practice. Such a choice creates phase reversal deflections in the EEG signals.

A simplified approach to this problem would describe the measuring of electrical activity for a *t* moment in time.

(11) $$\begin{array}{l} V_{AB}=-\oint_{A}^{B}E\cdot dl=-\frac{Q}{4\pi \epsilon }\left(1∕r_{A}-1∕r_{B}\right). \end{array}$$

where *E* is the electric field created by a point charge Q, at a distance *r*_*A*_ and measured in respect to the electric potential at a distance *r*_*B*_. In order to adequately measure *V*_*A*_ in a way that its value can be used for reference purposes a position must be chosen where the potential is close or equal to zero and more critically *constant* over time.

(12) $$\begin{array}{l} V_{AB}=V_{A}-V_{B}=V_{A}-0=V_{A},\;as\;r_{B}\to \infty \text{ then }1∕r_{B}=0 \end{array}$$

However, if the reference electrode is placed near electrically active areas i.e., within the EEG sensor array it is rather prone to change its potential value over time. More specifically, when a high in amplitude EEG element *V*_*event*_(*t*) takes place, the reference electrode's initial *V*_*B*_ value is shifted to $V_{B}^{\prime }$ (Equation 13) affected by the volume conducted *V*_*event*_(*t*):

(13) $$\begin{array}{l} V_{B}^{\prime }=V_{B}+\;\frac{1}{e^{x}}V_{event}\left(t\right)\neq constant,where\;x\\ \;=\left|distance\left(reference-event\right)\right| \end{array}$$

Consequently, all channel measurements of the EEG are being affected by a phase reversed *V*_*event*_ value by being measured with respect to $V_{B}^{\prime }$ (Equation 13) essentially in relation to each sensor's distance from the element.

Artifactual values caused by poor choice of reference are simultaneous and have opposite polarity (Equation 14) to the original EEG element due to volume conduction and in relation to the symmetry of electrode array that is defined by the position of the reference electrode.

(14) $$\begin{array}{l} \varphi_{reflection}=-\pi \end{array}$$

The imaginary part of a complex number such as coherency is zero for −π (180°). Hence, it can be speculated the suggested methodology is not prone to the EEG reference problem. This is further demonstrated by simulation signals shown in the results section.

#### Statistical significance and error assessment

True coherencies can never be granted for experimental data related to unknown probability density functions of associated stochastic processes (De Munck et al., 1992; Nunez et al., 1997) and only estimates are possible. Here we seek for an accurate model to assess errors, not by making assumptions that relate to the probability distribution of the noise process, but rather by simulating the real acquired data in order to answer inferential questions and detect meaningful signal activity buried in the noise of unknown distributions.

A bootstrap-based approach is being employed here (Zoubir and Boashash, 1998; Zoubir and Iskander, 2007) designed for the evaluation of significance for EEG-element connectivity that approximates the distribution function of our estimator originating from the original data:

For the frequency data of a κ element epoch *Sx*(*f*), *Sy*(*f*) calculated from the collected EEG data, random samples *Sx*^⋆^(*f*), *Sy*^⋆^(*f*) are drawn using a pseudo random number generator with replacement from *Sx*(*f*) = {*d*_*x*_(*f*, 1), …, *d*_*x*_(*f, n*)} and *Sy*(*f*) = {*d*_*y*_(*f*, 1), …, *d*_*y*_(*f, n*)} of *n* periodograms. The resampling by replacement takes place over all selected κ element trials in a 1-by-1 scheme, populating the model with a full set of resampled trials that originate from each collected element in order to simulate precisely the calculation and characteristics of the proposed connectivity estimator. *Z* ${\text{Coh}}_{yx}^{⋆}\left(f\right)$ bootstrap statistics derive from a large *Z* number of repetitions of the above procedure. For the confidence interval estimation of a given *a* level of significance, where $P_{\left(C_{l}(|C_{YX}(\omega ){|}^{2})\leq |C_{YX}(\omega ){|}^{2}\leq C_{u}(|C_{YX}(\omega ){|}^{2}\right)}=1-a$, the percentiles of the ordered distribution of all bootstrap estimates are calculated.

Based on substitution and simulation principles, we suggest, that this model can adequately answer inferential questions: we determine the confidence bands and adjust to zero all values of our original estimates that could possibly (α-level) occur randomly (null hypothesis), as approximated by the above procedure. The bootstrap approach to similar applications has recently been proven to be more accurate (Zoubir and Iskander, 2007) than other (Wang and Tang, 2004) and well-established methods (Enochson and Goodman, 1965), including large coherences and non-Gaussian data.

#### Visualisation of results

The estimation of bivariate interactions between electrical activities acquired by distinct scalp regions is presented in connectivity maps showing values of interaction over frequency and time domains for all possible combinations of selected scalp electrodes. The above allow the detailed investigation of possible alternation of patterns for different frequencies and over time.

More specifically in the connectivity maps, each point of the x and y axes represents a specific scalp electrode location of the EEG recording system. Each box represents connectedness between two different scalp electrode locations, as labeled above each box. x and y axes of each box represent time and frequency respectively. Time and frequency limits are set according to the time-frequency plots of the sleep spindles (Table 1). Levels of connectedness are shown in relation to the color intensity with different shades of either red or blue (values shown in color bar). Furthermore, positive and negative information flow is represented in warm (red) and cold (blue), respectively. Taking the example of a box labeled as “F1-F2,” red color refers to high level of interaction between the two regions with the information flowing from the F1 toward F2 scalp electrode locations, whereas blue color stands for high level of interaction with the information flowing from the F2 toward F1.

**Table 1:** **Fast sleep spindles oscillatory frequency characteristics**.

	**Subject 1**	**Subject 2**	**Subject 3**	**Subject 4**	**Subject 5**
Sex	Female	Female	Male	Male	Female
Age	27	26	24	27	33
Number of fast spindles	120	279	150	109	240
Frequency of maximal power (Hz)	15.20	13.45	14.05	13.15	14.55
Lower frequency limit (Hz)	14.00	12.30	12.80	12.05	13.45
Upper frequency limit (Hz)	16.45	14.65	15.30	14.30	15.70

### Subjects and procedures

Five individuals (3 females) aged between 24 and 33 years volunteered to participate in this study. All participants were good sleepers, without any difficulties in falling or remaining asleep during the night. They were all in good health and free from medication at the time of study. None of the subjects reported a history of neurological or psychiatric disorder, or disordered sleep. Subjects kept a 7-day sleep diary, and were instructed to follow their regular sleep schedule, and refrain from alcohol and caffeine at least 3 and 1 days respectively, prior to the experiment. Menstrual phase was not controlled for the female subjects. All participants read and signed an informed consent form, which described in detail the procedures and purposes of the study. Subjects were instructed to arrive at the laboratory ~1 h prior to their usual bedtime, the latter calculated as a 7-day bedtime average based on their sleep diaries. Each of them spent one whole night in the laboratory, in an air-conditioned soundproof temperature controlled dark Faraday-cage room that was intentionally not monitored to avoid potential sleep disturbances owing to the feeling of being watched. No pharmacological substance was used to induce sleep. Before sleep, subjects were instructed to keep their eyes closed and relax for a period of 2 min. Night sleep recording began after the subjects willingly switched off the room lights, as were instructed to do when they felt like falling asleep, and ended with their spontaneous wake up in the morning. Electrophysiological signals were monitored in an adjacent room and overnight communication with the subjects was established vocally through a microphone–speaker console system. In the morning, all subjects reported to have had a comfortable and undisturbed sleep.

All procedures described were approved by the University of Patras Committee for Ethics in Research.

#### Recording

All-night sleep was recorded using 58 EEG tin electrodes according to the extended international 10–20 system (FP1, FPZ, FP2, F3A, F4A, F7, F5, F3, F1, FZ, F2, F4, F6, F8, C5A, C3A, C1A, CZA, C2A, C4A, C6A, T3, C5, C3, C1, CZ, C2, C4, C6, T4, T3L, TCP1, C3P, C1P, PZA, C2P, C4P, TCP2, T4L, T5, P5, P3, P1, PZ, P2, P4, P6, T6, CB1, P3P, P1P, PZP, P2P, P4P, CB2, O1, OZ, O2) using an electrode cap (ElectroCap International, Eaton, OH, USA), that provided inter-electrode spacing of 4.5 cm. EEG electrode inputs were ear lobe referenced and grounded over the FZA position. A bipolar derivation of oblique electrooculogram was used to detect eye movements, for which electrodes were placed 1 cm above the right outer cantus and 1 cm below the left outer cantus, and a bipolar EMG from the upper masseter muscle was used to track muscle tone changes. Impedance of all electrodes was kept below 10 kOhms for most of the night. Physiological parameters were AC recorded, amplified at a total gain of 1000, band-pass filtered at 0.05–500 Hz and digitized through a 16-bit resolution A/D converter, which provided an accuracy of 0.084 uV/LSB, at a sampling frequency of 2500 Hz by a Synamps system (Neuroscan, Charlotte, NC, USA), and stored on hard disk. The 50-Hz notch filter was not applied during recording. Subject movements during sleep were detected by a sensitive motion detector placed over the bed area that produced a 2-s Transistor-Transistor Logic signal every time movement occurred. The motion detectors signal was recorded as an external trigger and was stored along with the electrophysiological signals as an event channel.

#### Scoring and selection

Manual sleep staging was performed by visual inspection of the EEG recordings along with EOG and EMG channels using the criteria of Rechtschaffen and Kales (1968), taking into consideration the propositions of the AASM Visual Scoring Task Force (Silber et al., 2007) and the DGSM Task Force (Rodenbeck et al., 2006), and keeping a time resolution of one second.

The sleep spindle was identified as a >500-ms train of 11–16-Hz waves. Fast sleep spindles were identified according to the definition of Gibbs and Gibbs (Gibbs and Gibbs, 1950). Fast spindles (>13 Hz) exhibit a symmetric bilateral distribution over centroparietal areas. All of the fast spindles were selected from only NREM stage II periods of the whole-night sleep of our subjects. All of the selected fast spindles were preceded and followed by at least 1 s of silent EEG background i.e., not preceded or followed by any external events or EEG elements (K-complexes, Vertex waves, delta waves etc.).

Sleep staging and scoring were performed and validated across 3 independent reviewers. Sleep staging and scoring was performed manually rather than automatically in order to provide with overall proof of concept for the methodology.

#### Analysis

Manual cursor marking offered by Scan software (Neuroscan, Charlotte, NC, USA) was used to create event channels. NREM stage II epochs from the whole night sleep recording and free of any movement and other artifacts were selected. Precise time-markers were then placed over the most prominent first, middle and last negative peak of the oscillatory elements under study. For all markers, the peak was marked over the record of the Cz electrode, where fast spindles are prominent.

Event-related data were further processed by a custom-made MATLAB-based (The Mathworks, Natick, MA, USA) software suite developed at the Neurophysiology Unit. FFT-based event-related time-frequency analysis was performed for each selected element within a time-window centered (time = 0.00) at the marked element, from 0.05 to 20 Hz at a step of 0.05 Hz. More specifically, fast spindles were analyzed in a time-window of −1.00 to 2.5 s. Fine analysis resulted in averaging the time-frequency plots (**Figure 3**) for all trials of each subject. The power spectral density (PSD) values appear as the dB magnitude of the spectral analysis that is as 10 × log(10) [POWER(Uv^∧^2)] over the selected time interval. No filter was applied to the processed electrophysiological data. All frequency and PSD values were measured over the PZ electrode unless otherwise stated.

The connectivity maps were estimated by the use of a custom-built MATLAB-based tool featuring the suggested methodology, which combines specific techniques at the six levels of pre-processing, correlation, and directionality estimation, control of EEG volume conduction and reference problems and statistical analysis.

## Results

### Evaluation of techniques

As a proof of concept, we examined coherence on computationally generated signals prior to application on real EEG data. For a set of time series with known characteristics we tested the methods with regards to the parameters of phase (see Appendix) and number of trials under calculation and moreover with regard to the volume conduction and reference electrode problem. In Appendix, we present further tests on the detection of correlations between simultaneously occurring signals with various frequency components (see Section Coherence for Zero Lag Correlations and Coherence for Signals with Different Frequencies in Appendix) and signals with a constant time shift (see Section Coherence for Non-zero but Constant Lag Signals in Appendix).

We define here as *x, y, z* sinusoid time series of 14, 14, and 15 Hz frequency, respectively. Random noise of relatively low amplitude is added to *y, z*. All signals consist of 2500 samples per second.

For the calculation of coherence estimates, the short-time Fourier transforms using Hamming window of 2048 samples with 2000 overlap and 0.05 step is applied.

#### Mean magnitude squared coherence over κ trials

**Figure 1:**
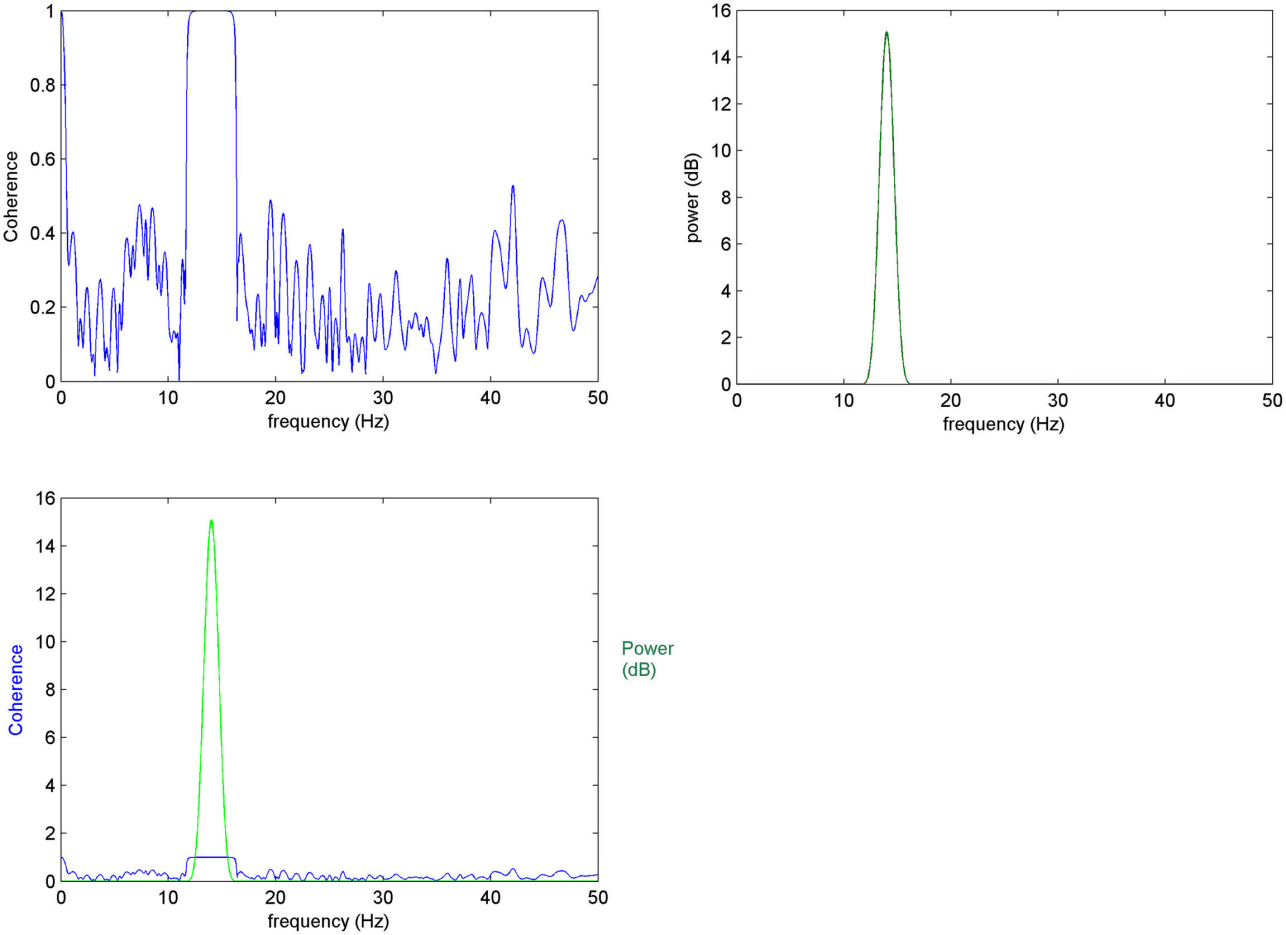
**Mean coherence in *K*-trials: (Top left) Coherence between 10 trials of *x* and *y* signals, (top right) power spectrum of *x*, (bottom left) top images compared: it is expected for coherence changes to take place at frequencies that characterize the elements of interest**.

It has been shown (Nunez et al., 1997; Appendix A in Supplementary Material) that Coherence is able to detect correlations of a pair of signals in the frequency spectrum. However, as discussed in methods, estimates can take conditionally high values for non-true frequencies (**Figure A1**). We demonstrate here coherence behavior when multiple κ trials (Figure 1, Figure A1) are taken into consideration.

When mean coherence is calculated for *K* = 10 trials of *x* and *y* the variance of coherence is significantly reduced shifting non-meaningful (i.e., non-true) coherencies to zero. This improved result is due to the averaging and normalization over a greater number of trials, as expected by the equation of normalized RMS error (Equation 6).

Thus, as trials increase in number, the non-meaningful values of mean coherence decrease making the estimates more accurate. In this way, the proposed EEG-element methodology calculated over a large *K* number of selected element trials (see Section Prepossessing for the Element Coherence Estimation) gives results supported by robust descriptive statistics while further allowing the estimation of coherence with greater resolution over time and frequency domains.

#### Reference electrode and the imaginary part of coherence

The Imaginary part of coherence is not subject to artifactual phase-reversed potentials related to defective positioning of the reference electrode (see Section Methodology):

For sinusoid signals (Equations 15–17), generated accordingly in order to simulate the EEG reference problem, the Coherence and Imaginary Part of Coherence between y–x and z–x time series are being calculated.

(15) $$\begin{array}{l} x\left(t\right)=10sin\left(2\pi ft\right),\;where\;f=14Hz \end{array}$$

(16) $$\begin{array}{l} \left(t\right)=x\left(t+\varphi_{1}\right)\;+noise,\;\varphi_{1}=\pi \end{array}$$

(17) $$\begin{array}{l} z\left(t\right)=x\left(t+\varphi_{2}\right)\;+noise,\;\varphi_{2}=\pi ∕4 \end{array}$$

**Figure 2:**
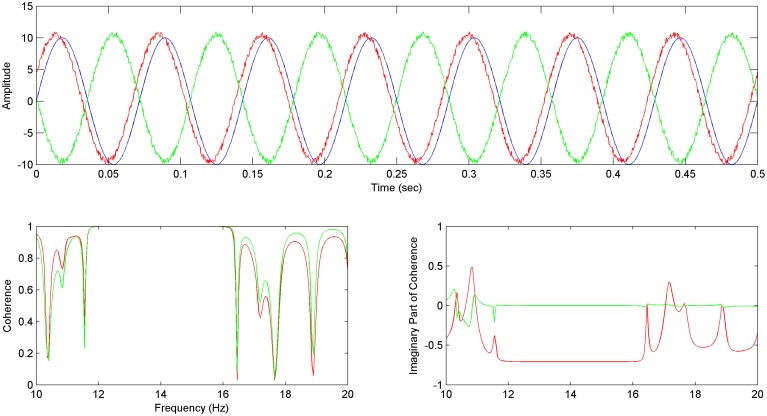
**Imaginary Part of Coherence and the reference problem: In the above image (top) the *x* (in blue), *y* (in green), *z* (in red) sinusoid time series are shown, with *z* preceding *x* and *y***. (Bottom left) Coherence between *y* and *x* C*yx* (**Δ**φ = π) (in green) is almost equal to the Coherence between *z* and *x* C*zx* (Δφ = −π/4) (in red). (Bottom right) The Imaginary Part of Coherence between *y* and *x* IC*yx* (Δφ = π) (green) gets zeroed for correlated frequencies due to the phase shift of Δφ = π which has been described as a common characteristic of the EEG reference problem effect (see Section The EEG Reference Problem). The Imaginary Part of Coherence for *z* and *x* pair IC*zx* (φ = −π/4) (in red) takes high negative values for correlated frequencies, characterizing the preceding of *z* with regards to *x*.

We hereby, show that by using the Imaginary part of coherence with the proposed methodology, faulty values addressed to the EEG reference problem get adjusted to zero (Figure 2 bottom right), while directionality is being addressed by the use of the phase, which essentially gets calculated in the estimation of coherence (Figure 2 bottom right).

### Connectivity patterns of the sleep spindle

We note here, that it is meaningful to initially observe connectivity values for frequencies that characterize the elements of interest (Figure 1 bottom).

For five healthy volunteers the first peak of each sleep spindle that occurred at the second stage of sleep has been manually annotated. We measured the characteristic frequency band of the sleep spindles of each subject using the average spectrogram of all annotated sleep spindles.

#### Time-frequency analysis and characteristic frequency bands

**Figure 3:**
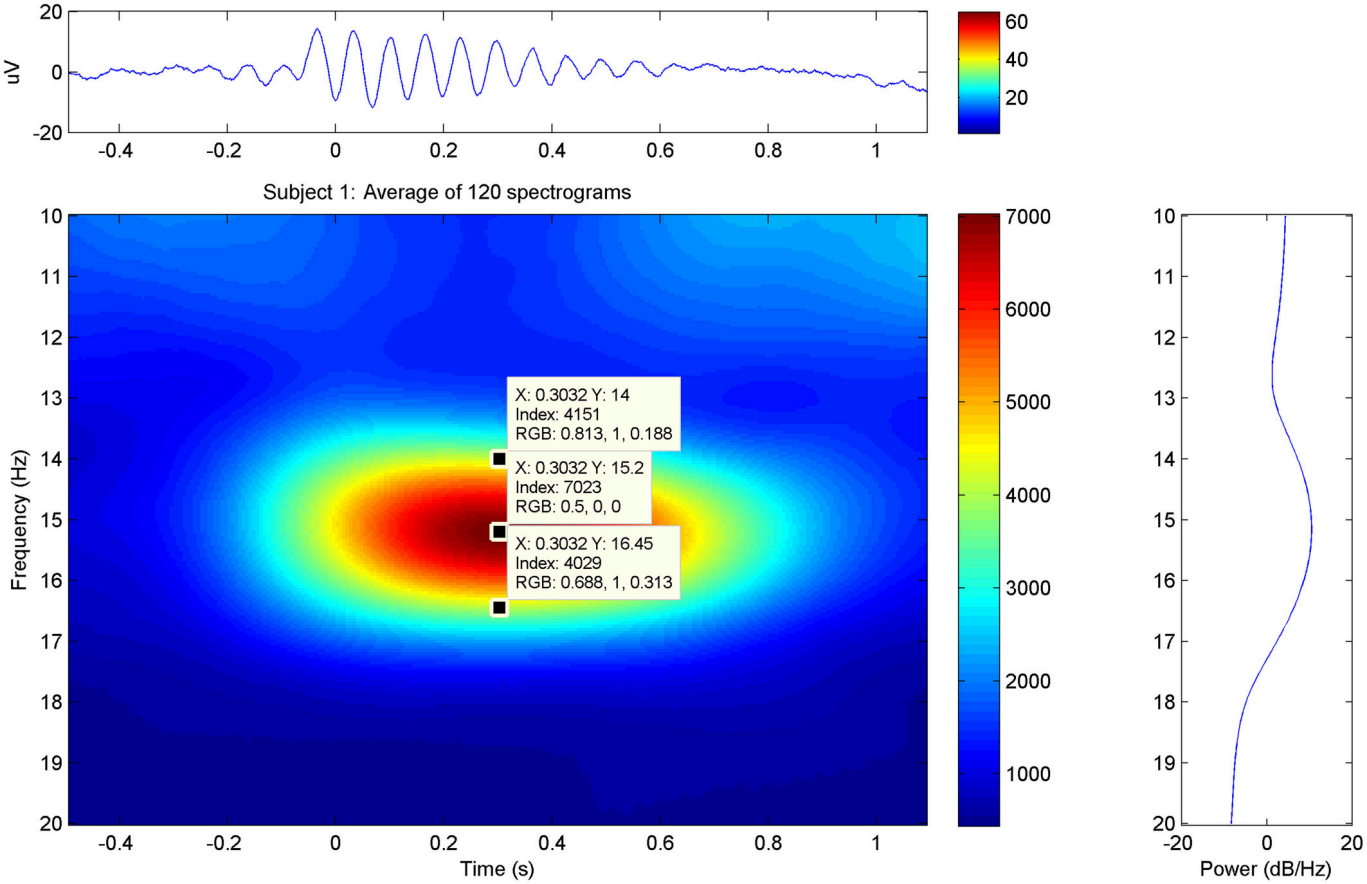
**Sleep Spindle Spectrogram: average spectrogram of sleep spindles for subject 1 in the PZ electrode**. The maximum and approximate upper-lower power changes have been manually annotated in the graphs.

In the average spectrograms of the sleep spindles of each subject data-tips were placed at the maximal, upper, and lower frequency limits (Table 1) according to significant power changes per color-bar (Figure 3).

#### Estimation of connectivity in fast sleep spindles

The estimation of connectivity patterns characterized by the sleep spindle for all possible pairs of electrodes is estimated using the proposed methodology via a custom MATLAB-based tool. The level of significance has been set at α = 0.05 with *Z* = 1000 number of bootstrap resampled estimates approximation.

#### Connectivity maps of healthy subjects

Connectivity maps for all subjects suggest the posterior and frontal regions to be strongly interacting when the fast sleep spindle function is on going, with all subjects showing the involvement of similar scalp-EEG electrodes (**Figure 7**). More specifically the connectivity maps showed interactions with prominent involvement of the OZ/P3/PZ/P4 and F3/FZ/F4.

**Figure 4:**
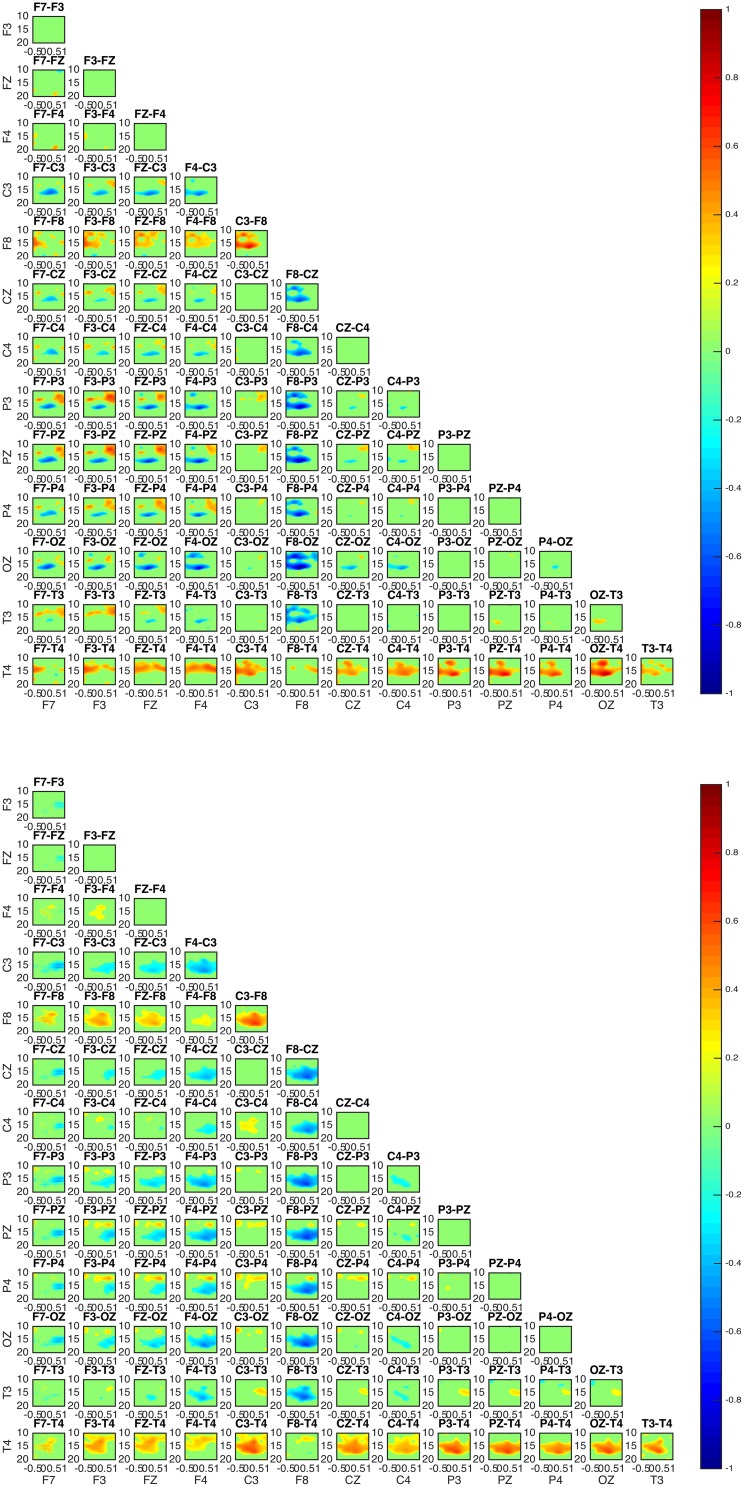
**Connectivity patterns: connectivity maps for subjects 1 (upper) and 5 (lower)**. The patterns between the two subjects appear almost identical while matching the patterns of subject 3. In all subjects 1, 3, and 5 the posterior regions appear to be causal to the frontal.

**Figure 5:**
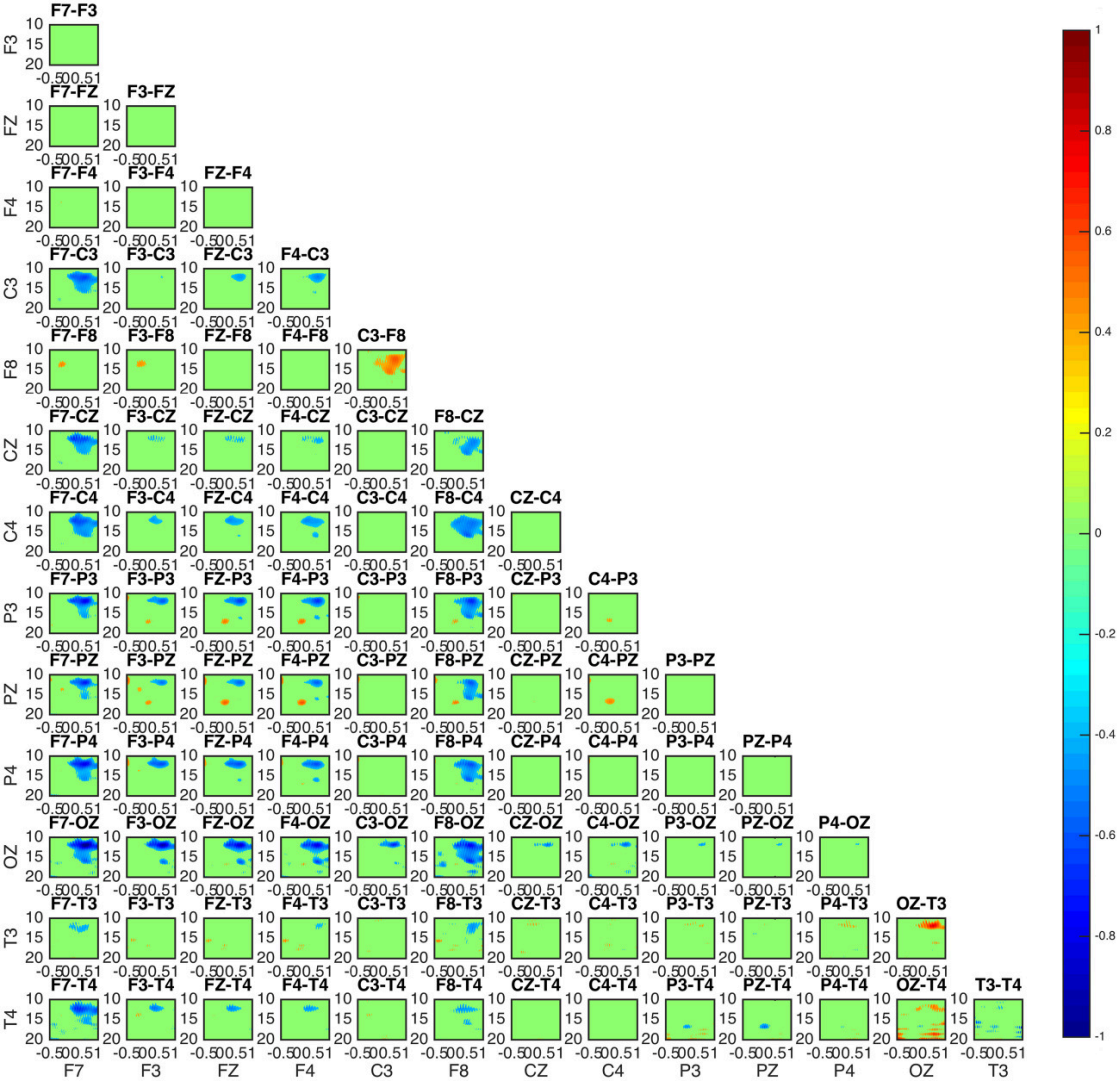
**Connectivity map of subject 3 appears to be similar to the patterns of subject 1 and 5 in Figure 4**.

Two groups were formed according to the directionality of information flow. In subjects 1, 3, and 5 show high interaction levels appear between posterior and frontal areas, with information flowing toward the latter (Figures 4, 5). Subjects 1 and 5 interaction and information flow patterns appear to be almost identical. Moreover, the regions that relate to the F8–T4 and OZ areas appear to be strongly interconnected.

**Figure 6:**
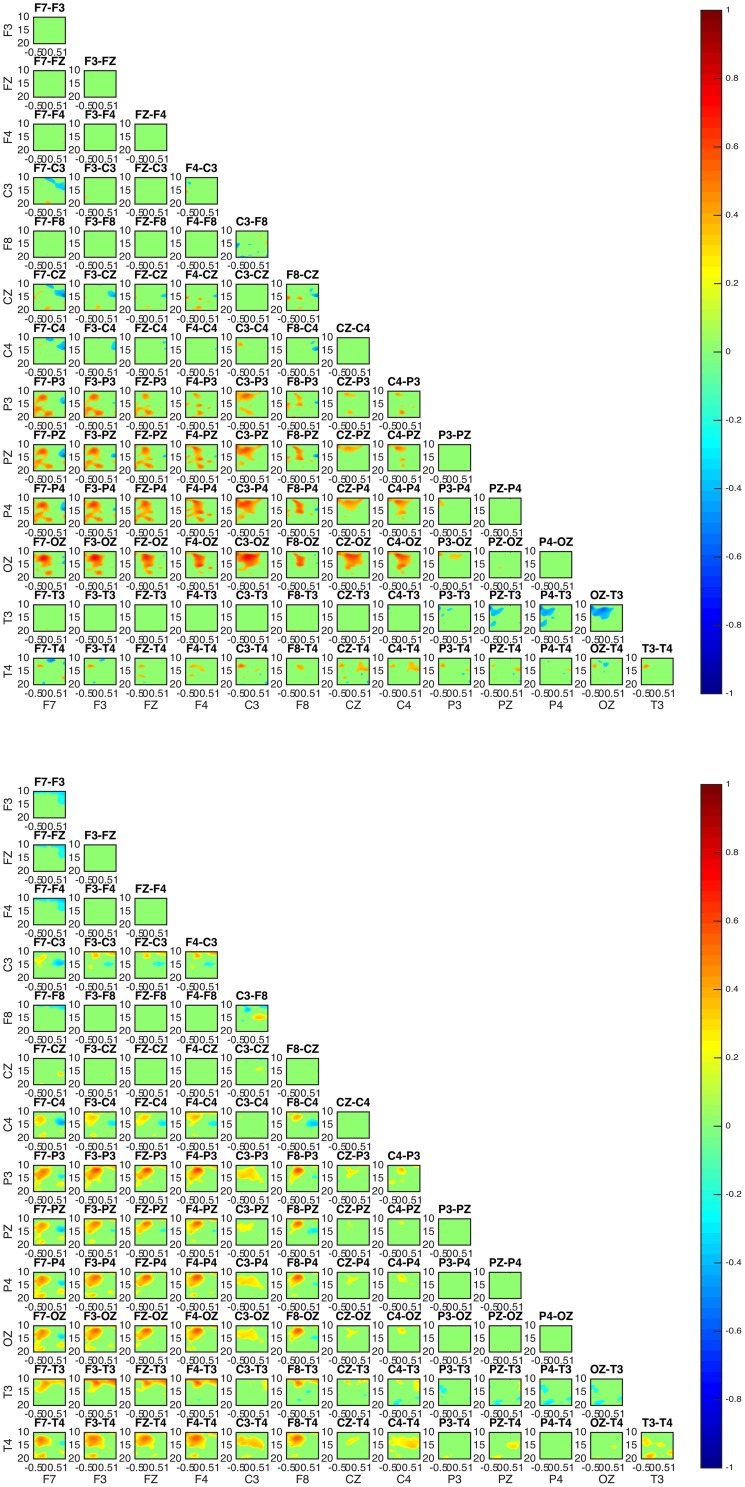
**Connectivity map for subjects 2 (upper) and 4 (lower)**. The involvement of areas similar to the ones in subjects 1, 5, and 3 is evident. For both subjects 2 and 4, OZ appears to be a strongly interconnected area. With regards to directionality, the information appears to move from frontal toward posterior areas.

The interaction patterns in subjects 2 and 4 appear to be very similar (Figure 6). A strong interaction pattern between centroparietal and frontal regions is revealed, with the information flowing from frontal to posterior areas. The area related to the OZ electrode appears to be strongly interconnected.

**Figure 7:**
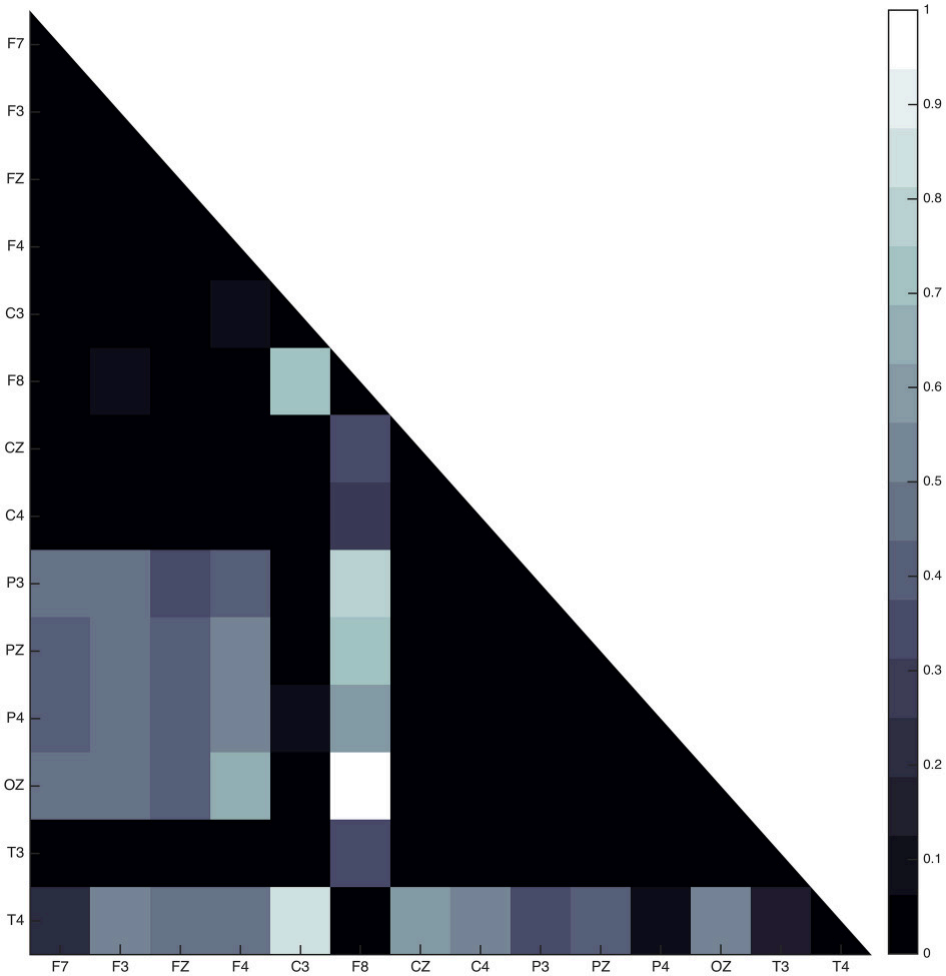
**Average absolute connectivity pattern across all subjects: the occurrence of sleep spindle significantly connects posterior and frontal areas**. The existence of three highly interconnected nodes - OZ, T4, and F8, is also evident.

In all subjects, the sleep spindle appears to significantly increase the connectedness of regions with a prevailing pattern (Figure 7) and three highly interconnected nodes (OZ, F8, T4) have been revealed.

Although the connectivity regional interaction patterns are similar in all subjects a clear differentiation between subjects is revealed with regards to the directionality of information flow. In subjects 1, 3, and 5 the information appears to mostly flow from centroparietal (OZ, P3, PZ, P4) toward frontal (F3, FZ, F4) and left frontotemporal regions (F8, T4). Conversely, in subjects 2 and 4 centroparietal regions (OZ, P3, PZ, P4) receive information from centro-frontal areas (F7, F3, FZ, F4, F8).

## Discussion

### Summary

In this study we suggest a methodology for the connectivity investigation of EEG microstructural elements. We evaluated its qualities via artificially generated signals and proceed with its application on microstructural sleep elements using the proposed scalp-EEG connectivity methodology. We address the problems of EEG reference and volume conduction while estimating the bidirectional interregional interaction levels over time and frequency.

### Details

We approach the estimation of interregional aspects that characterize this sleep phenomenon from a frequency-oriented angle (Wendling et al., 2009). Firstly, we describe the techniques of the methodology. We test the latter on artificially generated signals with regard to the volume conduction problem, the reference electrode problem and information flows. Finally, we calculate and present preliminary results for the connectivity patterns of the fast sleep spindle as acquired from the whole night sleep EEG recordings of five healthy subjects.

### Methods discussion

Coherency is a linear measure that detects correlations in the frequency spectrum for a pair of signals. Considering a system as stationary, the cross-spectrum on which coherency mostly relies on completely determines the dynamics. DTF (Baccalá and Sameshima, 2001) that can be considered a generalized approach to Granger Causality (Kamiñski et al., 2001; Chávez et al., 2003; Hesse et al., 2003) is vulnerable to additive noise, while the direction of information flux estimated from the asymmetry of Granger causality can also be sensitive to asymmetric noise levels (Nolte et al., 2004).

It has been shown in the past (Quian Quiroga et al., 2002) that the performance of other synchronization measures outside of coherence, namely non-linear interdependencies, phase synchronizations, mutual information and cross-correlation do perform in the same way and are valuable in the study of brain processes complementing the conventional visual inspection of the EEG. Here, coherence has been chosen for its ability to trace synchronizations limited to specific frequency bands, something of great importance in the connectivity characterization of EEG features with specific frequency signatures.

The involvement of thalamus in the generation of sleep spindles via thalamocortical circuits is widely known. It is difficult to infer anything about interactions between the cortex and deeper brain structures by the use of scalp EEG recordings alone. Here we focus on the characterisation of networks that correspond to the scalp-EEG electrode space. Differences between the coherency estimates at scalp electrode space and at intracranial source level are unavoidable but do not necessarily invalidate EEG cognitive or medical studies of robust coherency changes Nunez et al., 1997. However, connectivity findings at the EEG electrode space can generally suggest gross potential relationships and therefore, hypotheses related specifically to brain regions have to be further assisted by analysis at the level of brain sources i.e., by MEG estimations (Zeroualli et al., 2014) or intracranial recordings (Frauscher et al., 2015).

The study of well-specified EEG elements allow us to calculate coherency in an advantageous way allowing for better accuracy and time-frequency resolution.

The Imaginary part of Coherence was chosen as the coherency estimate since it has been shown to be able to eliminate ‘self-interaction’ values caused by volume conduction and estimating information flow through phase lags among the time series. We show here how it could possibly be able to better interpret connectivity by potentially eliminating correlation values related to the EEG reference problem.

A bootstrap approach was designed for the statistical analysis. The latter is proved to be a powerful method of modeling since it offers better accurate than other (Wang and Tang, 2004) and well-established methods (Enochson and Goodman, 1965), including large coherence and non-Gaussian data (Zoubir and Iskander, 2007).

#### Statistics and error assessment

Coherency is a method featuring statistical normalization with respect to the diagonal elements of cross-spectrum. We have shown that coherency values not related to the element of interest drop with the increase in number of trials. This study's results, rely on the robust coherence changes due to the occurrence of fast sleep spindles and the clear regional patterns revealed.

Application of inappropriate statistical tests can result to erroneous estimates (Nunez et al., 1997; Ioannidis, 2005; Lambdin, 2012). We strongly base our study on descriptive statistics (Trafimow and Marks, 2015) and by the usage of large sample sizes (i.e., trials of elements) we aim for increased accuracy in our methodology. Moreover, we apply a bootstrap-based analysis designed for the purpose of our study. Such statistical technique can be considered an accurate model for answering inferential questions by using the already acquired data in order to adequately simulate the systemic and neurophysiological element-related attributes of the EEG signals.

## Conclusions

Our results mainly correlate with the frequency signatures and the duration of sleep spindles suggesting similar regional network patterns for all subjects. In some subjects, a second network appears to get activated shortly after the occurrence of the sleep spindle for alpha band frequencies. This promotes the investigation of the proposed topographical maps across frequency and time domains outside of the bands that purely characterize the element under investigation, where we expect a network to form.

This methodology can be useful in the estimation of connectivity focusing patterns in any transient repeating EEG phenomena of short duration, such as sleep spindles, K-complexes or spike wave discharges. In particular, focusing on the sleep spindles paradigm, the high time resolution may allow the correlation of transient modulations in spectral frequency (shown by MEG studies to occur during the course of spindles) with the associated changes in EEG connectivity (Zeroualli et al., 2014).

## Author contributions

This research is conducted primarily by DS in partial fulfillment of the requirements of his Ph.D. thesis at the UoP. All authors have contributed to the recording of sleep EEG experiments, recruitment of subjects, writing and revising the manuscript. GK and MK guided and supervised the study. All authors designed the experimental design. DS, AK, and VK researched, tested, evaluated all techniques used for this study. DS, AK, and VK developed the computational tools for this study. All authors scored and cross validated the EEG recordings used in the study. Further, evaluation and development of the methods for the significance, statistical analysis, presentation of results as well as application of the methodology to real EEG recordings have been performed by DS.

## Funding

This study was supported by the ARMOR EU research project (European Commission under the Seventh Framework Program (FP7/2007-2013) agreement number 287720). AK has been co-financed by the European Union (European Social Fund – ESF) and Greek National Funds through the Operational Program “Education and Lifelong Learning” of the National Strategic Reference Framework (NSRF) – Research Funding Program: Heracleitus II. Investing in knowledge society through the European Social Fund.

### Conflict of interest statement

The authors declare that the research was conducted in the absence of any commercial or financial relationships that could be construed as a potential conflict of interest.

## Figures and Tables

**Figure A2:**
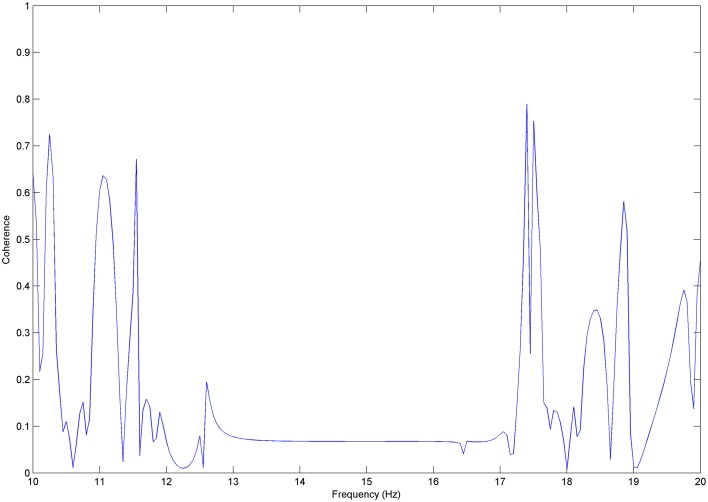
**Coherence for *x* and *z* pair: very low coherence values can be observed for the non-correlated frequencies, whereas coherence takes up to 0.8 values due to random correlations between the random noise and *x* in the frequency domain**.

**Figure A3:**
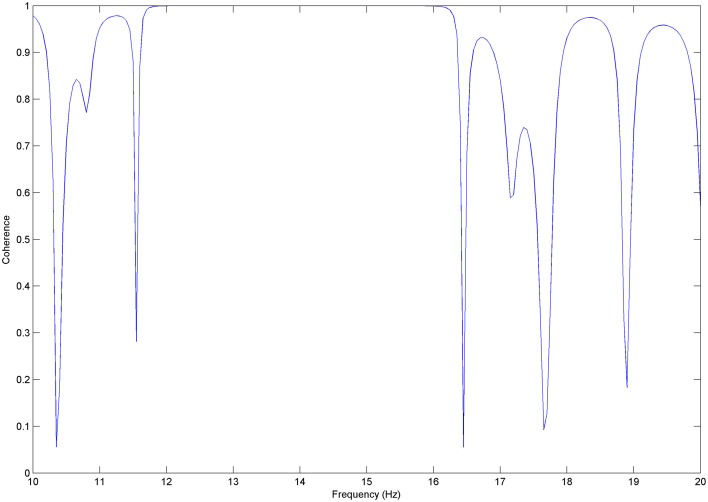
**Coherence and phase delay: coherence values between *x* and *y* signals time shifted by Δφ = π/4**.

## Appendix

### Coherence for zero lag correlations

We estimated the coherence values between an *x* sinusoid time series of 14 Hz frequency, *y* sinusoid of 14 Hz frequency with random noise of low power added to the second one. Both signals have 2500 samples per second (*fs* = 2500 Hz).

In order to estimate coherence, we calculate the short-time Fourier transform using Hamming window of 2048 samples with 2000 overlap and 0.05 step.

Coherence for the *x-y* pair for *k* = 1 trial, takes its maximum value (1) (Figure A1, left) for the frequency band of about 14 Hz whereas for the rest of the frequency spectrum, it fluctuates between 0 and 0.8 values. On the other hand, when *k* = 10 trials are taken into the calculation of coherence between *x* and *y*, the coherence values attributed to noise appear to be significantly reduced fluctuating between 0 and 0.3 (Figure A1, right).

It is critical to mention here that the activity of the two signals show no differences in phase as they occur simultaneously. We deliberately address zero-lag, i.e., synchronous correlated activity to the volume conduction effect.

### Coherence for signals with different frequencies

We test Coherence between *x* and *z*, where *z* a sinusoid time series of 15 Hz frequency and random noise of low power (Figure A2).

Coherence takes its minimum values for the frequency band about 14 and 15 Hz, where the two signals differentiate with regards to their frequency components. Outside that band coherence fluctuates between 0 to 0.8 values, which apparently can be addressed to similarities between *x* and the random noise.

### Coherence for non-zero but constant lag signals

We test coherence between χ and *y*, where *y* was steadily time lagged by Δφ = π/4. Coherence here takes its maximal value (1) (Figure A3) for the frequency band of about 14 Hz. We therefore show that coherence estimates the correlations of two processes that keep their time-phase constant, i.e., are phased-locked.
